# Case report: Complete paternal isodisomy on chromosome 18 induces methylation changes in *PARD6G-AS1* promotor in a case with arthrogryposis

**DOI:** 10.3389/fgene.2023.1297754

**Published:** 2023-12-21

**Authors:** Johanna Moch, Maximilian Radtke, Janina Gburek-Augustat, Maike Karnstedt, Senta Schönnagel, Stephan H. Drukewitz, Laura Pilgram, Julia Hentschel, Isabell Schumann

**Affiliations:** ^1^ Institute of Human Genetics, University of Leipzig Medical Center, Leipzig, Germany; ^2^ Division of Neuropaediatrics, Hospital for Children and Adolescents, University of Leipzig Medical Center, Leipzig, Germany

**Keywords:** uniparental disomy, allele frequency, imprinting, gene regulation, next-generation-sequencing, methylome, case report

## Abstract

Uniparental disomy (UPD) is the inheritance of both alleles of a chromosome from only one parent. So far, the detection of UPDs in sequencing data is not well established and a known gap in next-generation sequencing (NGS) diagnostics. By developing a new tool for UPD detection, we re-evaluated an eight-year-old individual presenting with scoliosis, muscle weakness and global developmental delay. Previous panel analysis identified a homozygous likely pathogenic loss-of-function variant in the *PIEZO2*-gene associated with arthrogryposis (OMIM # 617146). Interestingly, during a re-evaluation process, we identified a region of homozygosity (ROH) covering over 95% of chromosome 18. Segregation and microsatellite analysis within the family revealed that only the father is a heterozygous carrier of the variant in *PIEZO2* and confirmed paternal uniparental isodisomy (iUPD) on chromosome 18 in the individual. Further methylation analysis indicated demethylation of the promotor region of *PARD6G-AS1*, which is described to be maternally imprinted and could possibly influence the individuals’ phenotype. Our report describes the first complete iUPD on chromosome 18 and highlights that UPDs can be a cause for homozygous pathogenic variants, which reduces the risk of reoccurrence in case of a new pregnancy in comparison to an autosomal recessive inheritance trait significantly.

## Introduction

PIEZO2-deficiency is a rare disease described in context of a distal arthrogryposis syndrome through loss-of-function variants on both *PIEZO2* alleles (OMIM #617146) (Szczot et al., 2021). *PIEZO2* encodes for a sensory cation-channel which is needed for mechanic sensations like proprioception, interoception and touch (Ranade et al., 2014). Most patients with PIEZO2-deficency present with muscle hypotonia, absence of tendon reflexes, scoliosis and motor developmental delay (Yamaguchi et al., 2019).

Homozygous variants can be caused by UPD, which describes the inheritance of two copies of a whole chromosome from only one parent. Whereas both chromosomes from one parental pair result in a heterodisomy (hUPD) and are caused by nondisjunction errors in meiosis I, the inheritance of two identical duplicated copies of one parental chromosome is called isodisomy (iUPD) and emerges of a nondisjunction error in meiosis II or a mitotic error (Benn, 2021). Possible mechanisms giving rise to UPD include monosomic or trisomic rescue, gamete complementation and postfertilization mitotic error (Eggermann et al., 2018). UPD can affect either the entire chromosome or only segments of it and depending on the inheriting parent maternal (UPD(mat)) and paternal (UPD(pat)) versions can be discriminated (Benn, 2021). UPDs are not mandatorily pathogenic and mainly cause symptoms if imprinting effects, chromosomal disturbances or homozygosity of recessive variants occur (Eggermann et al., 2018).

Within this report, we describe an eight-year-old individual with a homozygous variant in the *PIEZO2*-gene caused by a complete paternal isodisomy of chromosome 18 presenting with scoliosis, muscle weakness and global developmental delay. Furthermore, we were able to demonstrate a demethylation of the *PARD6G-AS1* promoter region, the consequences of which require further investigation.

## Materials and methods

### Genetic analysis

Genomic DNA was extracted from blood samples as previously described (Zacher et al., 2021). Enrichment and library preparation was performed using Nextera DNA Flex Pre-Enrichment LibraryPrep (*IDT for lllumina Nextera DNA UD Indexes*) and a TruSight One Sequencing Panel including 4,811 genes (lllumina, May 2014). Libraries were sequenced with 150bp paired end reads on a NextSeq550 system (Illumina, Inc., San Diego, CA, United States). Secondary analysis was carried out in Varvis (Limbus Medical Technologies GmbH, Rostock, Germany) according to GATK best practices for germline variants on hg19. The average coverage of target regions was 100x. The tertiary analysis was performed with the browser-based genomics evaluation software of Varvis. A homozygous nonsense variant in *PIEZO2* was identified and classified as likely pathogenic according to the guidelines of the American College of Medical Genetics (ACMG) (Richards et al., 2015). The identified variant was submitted to ClinVar (Variation ID: 976307).

### Re-analysis of NGS data, sanger sequencing and microsatellite analysis

Re-analysis of the case has been performed using our custom-made tool for UPD detection (altAF-plotter) (Radtke et al., 2023). To validate the result of the re-analysis, Sanger sequencing of the variant in *PIEZO2* as well as fragment analysis with five microsatellites from chromosome 18 were performed of the individual and the parents (Applied Biosystems 3,500 Genetic Analyzer, ThermoFisher Scientific, Supplementary Table S1).

### Methylation site analysis

Targeted methylation sequencing was performed using Twist Human Methylome Panel according to the manufactures protocol (TWIST Bioscience, South San Francisco, United States). Ultrasonic fragmentation of the DNA was conducted using a focused-ultrasonicator (ME220, Covaris^®^, Massachusetts, United States). The average coverage of targets regions was 120x. As control group, six blood samples from healthy individuals were selected. The raw fastq files were processed using the Nextflow pipeline nf-core/methylseq1.6.1 (Ewels, 2023). From the resulting bedGraph files, all sites with less than 10 alignments were removed. Only sites present in all samples were considered for the search of differentially methylated regions (DMR). Two-sided Crawford Howell *t*-test was used to assign *p* values to individual methylation sites. The sites were mapped to genomic windows of size 5,000bp with 2,500bp overlap. For all windows containing at least 25 sites, an aggregated *p*-value was calculated using Fisher’s aggregation method. All regions with a mean methylation difference of at least 15% compared to the control group and a *p*-value≤0.05 were more closely examined.

## Results

### Case description

The individual is the first child of healthy and non-consanguineous parents with a history unremarkable for genetic disorders. She was spontaneously born at term (gestational week 40 + 0) with a weight of 3700 g (p71), a length of 51 cm (p37) and a head circumference of 34 cm (p25). After birth she was admitted to the critical care unit for two weeks because of difficulties breathing and was nourished through a gastric tube until she was four months old. She presented with major muscle weakness, poor swallowing, an inspiratory stridor with tracheomalacia, abnormality of joint mobility especially in her ankles and a clubfoot. She did not show any facial dysmorphism. At 11 months, she reached the milestone of free sitting.

During her development, muscle weakness, especially in her lower limbs, was a main problem and her motor development including fine and gross motor skills was delayed. Furthermore, she was diagnosed with lower limb atrophy and absent reflexes of the lower extremity. Besides that the individual did not have any impaired proprioception and her sensibility was undisturbed on all sides. She has developed left accented lumbar scoliosis, kyphosis and has enuresis nocturna. In contrast, her cognitive and speech development is age-appropriate although her speech is described as “washed-out” and hard to understand. Her treatment didn´t include medication and consisted of physical and speech therapy.

The attending physicians suspected structural congenital myopathies or hereditary neuropathy and a muscular panel was perfomed outside our institution when she was three years old, which was referred to as unremarkable. When she was five years old panel diagnostics was expanded and a panel on connective tissue diseases and neuropathies was performed in which the homozygous likely pathogenic variant associated with arthrogryposis could be discovered. Additionally a MRI showed an extension of the central chanel in between cervical vertebrae three to six but was assessed as an incidental finding equally to supplementary neurophysiological diagnostics including sensory-evoked potentials which were found unspecifically conspicious by the attending neurosurgeons. Because of scoliosis, spirometry was performed at age six, which was unremarkable, but it was recommended to be repeated if the scoliosis should be progredient.

**FIGURE 1:**
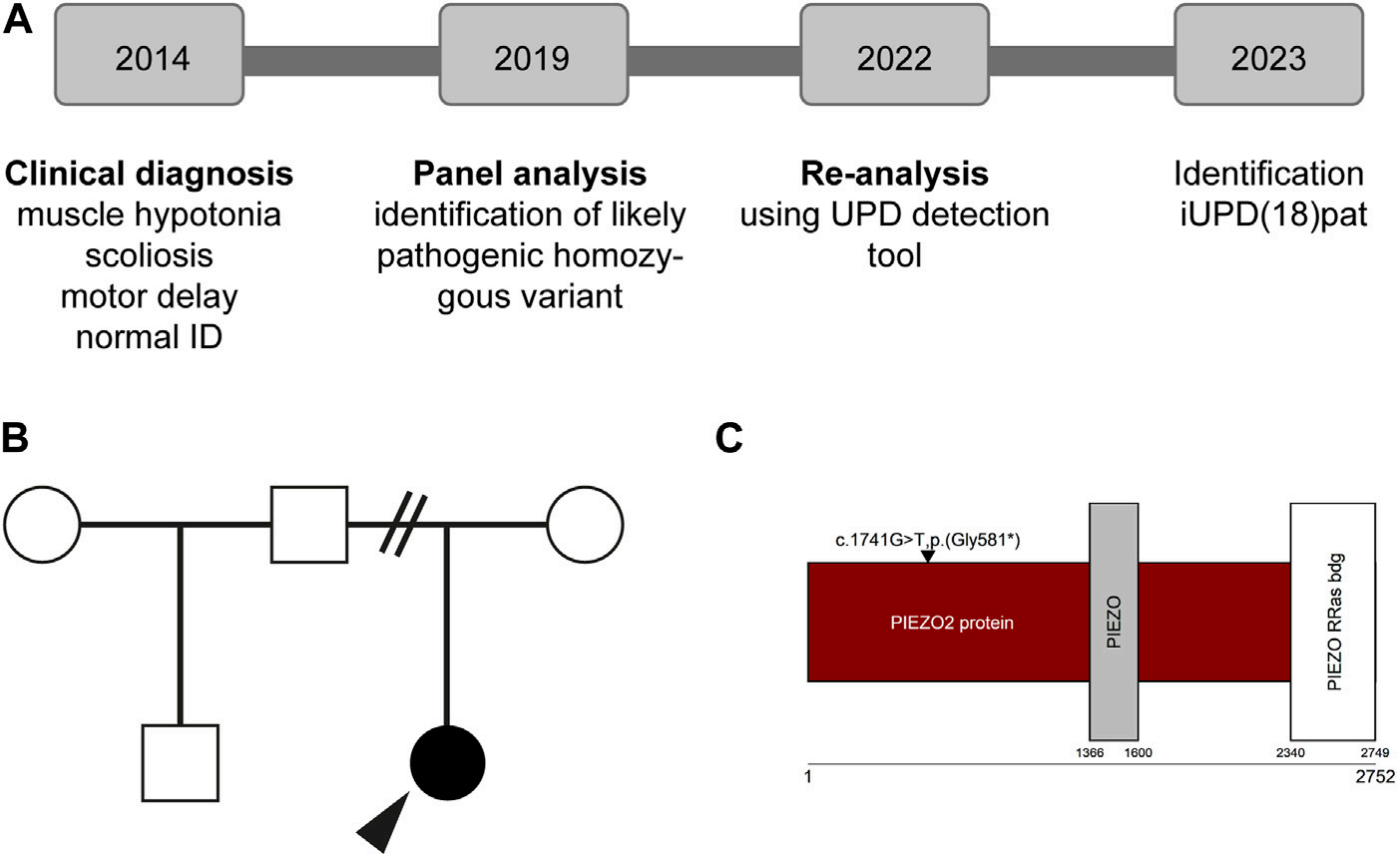
Overview of the individuals’ history **(A)** Diagnostic procedure including multigene panel analysis and re-evaluation of the data **(B)** Individuals pedigree **(C)** Protein and domain structure of *PIEZO2* (Uniprot, Q9H5I5). Note that the identified variant is not in a functional domain. ID; intellectual disability.

At age seven, her height was 117.5 cm (p14), her weight 22.3 kg (p37) and her head circumference 50.2 cm (p16). Her treatment still doesn´t include any medication and consists of different health promotions like physical and speech therapy, which seem to be well tailored for the patient. As aids, she uses whole leg orthoses, SDO orthoses, walkers with arm support, a wheelchair and an autoreha seat. She can pull herself up, walk 16 steps by herself and started an inclusive school. At age eight our UPD re-evaluation project took place and the parental isodisomy on chromosome 18 was discovered (Figures 1A, B).

### Re-evaluation NGS data

**FIGURE 2:**
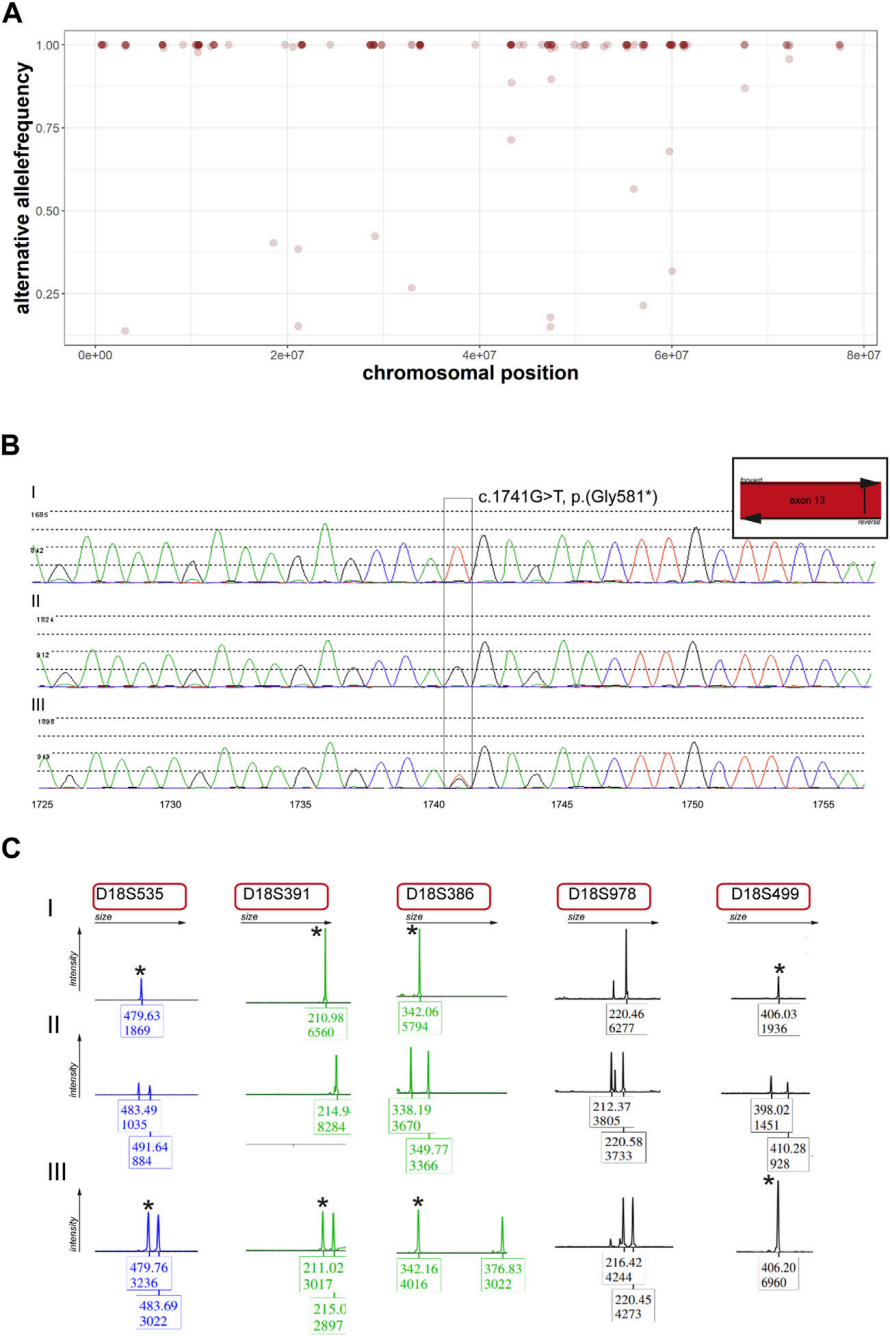
Re-analysis of panel data and segregation analysis within the individual’s family **(A)** Alternative allele frequencies of SNPs on chromosome 18 of the individual. Note the number of homozygous SNPs on the chromosome. **(B)** Sanger sequencing of the identified variant on *PIEZO2*. Note that the individual (I) is homozygous, the mother (II) does not carry the variant and the father (III) carries the variant in a heterozygous state. **(C)** Microsatellite analysis of five markers on chromosome 18. Stars indicate the same marker for individual (I) and father (III).

Panel analysis on neuropathy and connective tissue diseases has been performed when the patient was five years old. A homozygous loss-of-function variant NM_022068.3: c.1741G>T, p.(Gly581*) in exon 13 of the *PIEZO2*-gene was identified (Figure 1C). According to the guidelines of the ACMG (Richards et al., 2015) the variant was classified as likely pathogenic. Performing re-analysis using the altAF-plotter (Radtke et al., 2023) revealed that 96.47% of the individuals’ chromosome 18 is covered by regions of homozygosity (ROH). This suggests UPD to be a probable underlying cause (Figure 2A). Sanger Sequencing confirmed the variant in the individual and showed, that only the father was also a carrier (Figure 2B). Both parents do not show any similar symptoms. We assume that the father is a healthy carrier of the variant in *PIEZO2*. As the inheritance pattern is recessive and the fathers phenotype is unremarkable for arthrogryposis, we do not perform a new phenotyping of the father.

### Segregation and methylome analysis

**FIGURE 3:**
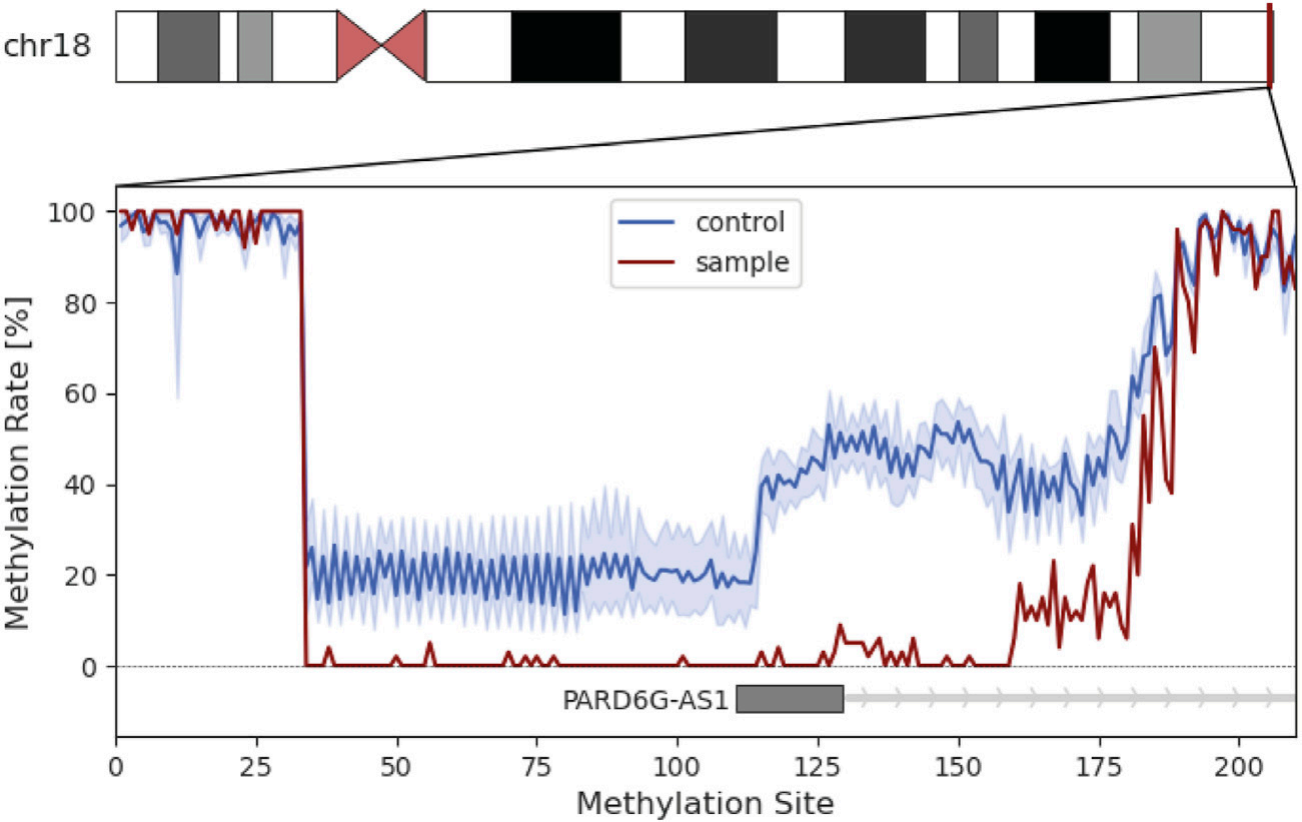
Methylation analysis of the individual’s chromosome 18. Insert represents the methylation rate in percent of the *PARD6G-AS1* promotor region. The methylation rate of the patient is shown in red. The average methylation of the control group is shown in blue with the standard deviation in light blue.

UPD validation was performed through microsatellite analysis. Out of five markers, four were informative for our hypothesis (Figure 2C). We revealed that the individual did not inherit any allele from the mother on chromosome 18 but shares those of her father. Methylome sequencing demonstrated demethylation of the potentially maternally imprinted promoter region *PARD6G-AS1* on chromosome 18 compared to control samples (Figure 3).

## Discussion

In a clinical multigene panel we found a homozygous loss-of-function variant in *PIEZO2* which leads to a PIEZO2-deficency-syndrome and is associated with distal muscle hypotonia, scoliosis and a global developmental delay (Szczot et al., 2021). After performing re-evaluation with altAF-plotter we assumed that the homozygous variant of the individual is caused by a paternal isodisomy of chromosome 18. Microsatellite analysis and Sanger sequencing confirmed our hypothesis of a paternal isodisomy. A possible mechanism explaining the UPD and homozygosity of the whole chromosome is a trisomic rescue which could have saved the individual from trisomy 18 (Edwards syndrome) and an even more severe phenotype. We assume that the maternal chromosome was eliminated resulting in a paternal uniparental disomy (Eggermann et al., 2018).

UPDs mainly cause symptoms in the context of imprinting effects, chromosomal disturbance or homozygosity for pathogenic variants (Eggermann et al., 2018). In order to not miss an imprinting effect, we have performed methylome analysis. Six genes (*IMPACT*, *BRUNOL4*, *FAM59A*, *ZNF396*, *TCEB3C* and *PARD6G*) with parent-of-origin-specific methylation on chromosome 18 are described so far (Zink et al., 2018), but only three of them (*ZNF396*, *TCEB3C* and *PARD6G*) are confirmed to have an imprinting effect (“Geneimprint: Genes” 2023). The identified hypomethylated promoter area of *PARD6G-AS1* is described as maternally imprinted and associated with multi-locus imprinting disorders (Sá Machado Araújo et al., 2018; Zink et al., 2018). An aberrant methylation pattern in *PARD6G-AS1* has been described in context of pseudohypoparathyroidism, Beckwith-Wiedemann-syndrome and transient neonatal diabetes as well as Edwards syndrome (Sá Machado Araújo et al., 2018). All of the above are not compatible with the individual’s phenotype, but since *PARD6G* might play a role in epithelial tight junctions, it is possible that a demethylation of *PARD6G-AS1* amplify it (Cunliffe et al., 2012).

To date, the incidence of UPDs is not conclusively known. Different studies found the UPD-incidence to be 1 in 2,000 in a healthy population (Nakka et al., 2019) or 3 in 1,000 in a patient cohort composed of trio-exome-sequencing data (Scuffins et al., 2021). Maternal UPDs seem to be three times more common than paternal UPDs (Nakka et al., 2019) which makes our case an unusual event. It has to be said that there are only a few studies reporting an UPD on chromosome 18 compared to the occurrence of UPDs of other chromosomes (Morgan et al., 2018; López-Garrido et al., 2022; Liehr, 2023). However, it is also possible that an UPD on chromosome 18 is not associated with a particular phenotype and thus, reports of this event are not available in the literature.

To summarize we reported a unique case of a homozygous variant in *PIEZO2* gene caused by a rare iUPD(pat) on chromosome 18. It is the first complete isodisomy described on this chromosome. So far, there is no specific therapy for *PIEZO*2-associated arthrogryposis but early interventions like physical therapy, speech therapy, orthopedic management and psychological education are important (Yamaguchi et al., 2019). Our finding is probably not going to influence the treatment of the individual but the reoccurrence rate of an isodisomy is negligible <1% (Erger et al., 2018), which is significantly different to 25% of an autosomal recessive inheritance trait. It is worth highlighting, that we could detect this UPD-case out of multigene panel data using our new tool altAF-plotter which is going to be published soon (Radtke et al., 2023). We highly advise considering UPDs as potential events leading to homozygosity, especially in families with no known consanguinity, as well as in cases that appear to have been resolved. Methylation analysis proposes that demethylation of *PARDG6-AS1* could strengthen the individual’s phenotype, but further functional studies are necessary to elucidate the influence of the altered methylation pattern of *PARDG6-AS1* on the phenotype described in this study.

## Data Availability

The datasets presented in this study can be found in online repositories. The names of the repository/repositories and accession number(s) can be found below: We have uploaded the data to https://www.ebi.ac.uk/ena/browser/home with the Study-Accession number PRJEB69274.
