# Supplementary material for: Case report: Complete paternal isodisomy on chromosome 18 induces methylation changes in PARD6G-AS1 promotor in a case with arthrogryposis
Source: Front Genet. 2023 Dec 21;14:1297754. doi: 10.3389/fgene.2023.1297754 (PMC10768175; doi:10.3389/fgene.2023.1297754)
Supplement: Supplementary file 1 [file Table1.DOCX]

Supplementary Material

# Supplementary Table

Table 1. Primer pairs for Sanger sequencing and fragment analysis

| Region | Forward | Reverse |
| --- | --- | --- |
| *PIEZO2*_Ex13 | TTGGGGTCTCCATCACTCCA | CTCTAAGCACCGCAAACTG |
| D18S535 | cagcaaacttcatgtgacaaaagc | caatggtaacctactatttacgtc |
| D18S391 | ggacttaccacaggcaatgtgact | ctggctaattgagttagattacaa |
| D18S386 | tgagtcaggagaatcacttggaac | ctcttccatgaagtagctaagcag |
| D18S978 | gtagatcttgggacttgtcaga | gtctcccatggtcacaatgct |
| D18S499 | agattacccagaaatgagatcag | gaaaatgtagaagtgagtcacct |

**
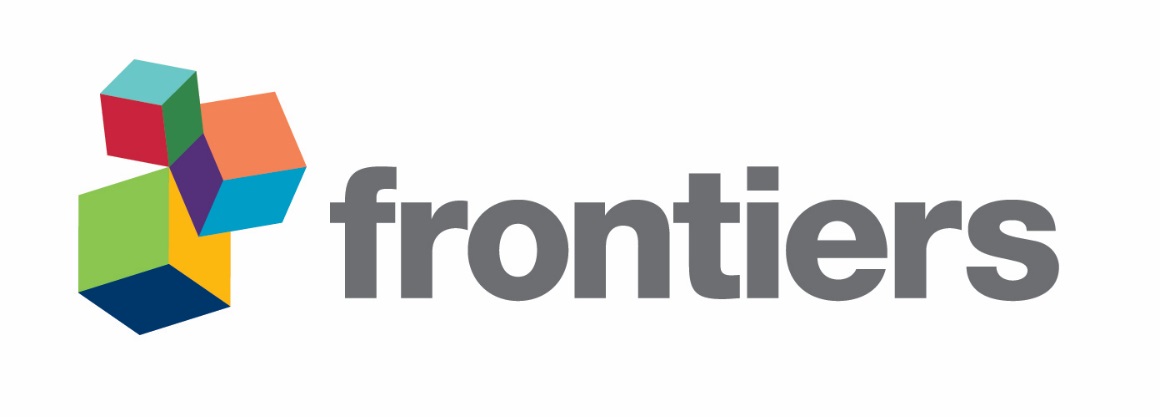
**
